# Analysis of the status of drug treatment in 746 inpatients with early-onset schizophrenia in China: a retrospective study

**DOI:** 10.1186/s12888-020-02962-w

**Published:** 2021-01-07

**Authors:** Jiuping Zhang, Xin Cheng, Huihui Zhang, Ping Xu, Peiying Jin, Xiaoyan Ke

**Affiliations:** 1 The Child Mental Health Research Center, The Affiliated Brain Hospital of Nanjing Medical University, GuangZhou Road 264#, Nanjing, 210029 China; 2 Department of Psychiatry, Nanjing Lishui Psychiatric Hospital, Nanjing, China

**Keywords:** Children and adolescents, Schizophrenia, Drugs, Status of treatment

## Abstract

**Background:**

There is limited evidence on the use of antipsychotics in patients with early-onset schizophrenia, which lags significantly behind the studies on adult patients’ medication and has a large disparity from actual clinical needs. Hence, this study aims to analyse the status of the drug use and its changes for patients with early-onset schizophrenia in our ward and to provide references on clinical medications for children and adolescents with schizophrenia.

**Methods:**

The distribution of antipsychotics on the day of discharge and their changes over time were retrospectively analysed in our inpatient department from March 2012 to July 2019. Descriptive statistical methods and *χ2* tests were carried out.

**Results:**

A total of 746 inpatients with early-onset schizophrenia were included. Among them, 99.3% of patients were prescribed atypical antipsychotic drugs, with 5.5% of patients prescribed typical antipsychotic drugs. The top five most commonly used antipsychotics were aripiprazole, olanzapine, risperidone, paliperidone and clozapine. Olanzapine and risperidone were used more frequently in men (*P* < 0.01), whereas aripiprazole was used less frequently (*P* < 0.01). Olanzapine and paliperidone were used more frequently in patients with adolescent-onset schizophrenia (AOS) (*P* < 0.05), and risperidone was used more frequently in patients with child-onset schizophrenia (COS) (*P* < 0.01). Multiple antipsychotics during hospitalization were prescribed in 23.1% of patients. The combination of aripiprazole and olanzapine was the most common in the AOS group, and the combination of risperidone and clozapine was the most common in the COS group. Before and after approval by the competent Chinese authorities, the use of paliperidone and aripiprazole tended to be stable.

**Conclusion:**

Atypical antipsychotics have been increasingly valued and used clinically. The consideration of medications for patients with early-onset schizophrenia needs to include factors such as age, sex, and severity of illness, metabolism and cognitive function at baseline.

## Background

Although early-onset schizophrenia has similar schizophrenic symptoms as adult-onset schizophrenia in terms of cognition, emotion, and perception, it also has own characteristics. Although the incidence of early-onset schizophrenia is low [1], hundreds of thousands of patients with early-onset schizophrenia can be found in China. Furthermore, the prognosis of children and adolescents is not as good as that of adult patients, with a lower response rate to treatment, a higher occurrence rate of mental disabilities and poor outcomes [2, 3]. Accordingly, the early detection and timely and effective treatment for patients with early-onset schizophrenia are particularly critical.

Antipsychotic medication remains the first-line treatment for schizophrenia. However, children and adolescents are in a special stage of growth and development, and many factors, such as efficacy, safety and acceptability, need to be considered during drug treatment. Atypical antipsychotics have proven to be safer and more effective, so they gradually have become the first-line drugs in the treatment of schizophrenia in children and adolescents [4, 5]. For example, a meta-analysis [6] that included randomized controlled studies between 1967 and 2017 showed that aripiprazole, olanzapine, risperidone, paliperidone and quetiapine have a significantly better therapeutic effect on patients with early-onset schizophrenia than placebo and that paliperidone, risperidone, olanzapine and quetiapine are somewhat better accepted than placebo. However, an increased risk of metabolic syndrome still exists in patients who are using atypical antipsychotics [7]. Ray, et al. [8] found that high-dose antipsychotics were associated with increased accidental mortality in children and adolescents. More importantly, few antipsychotics at present have been approved to be used in children and adolescents, which has led to off-label use in treatment [9].

At present, little solid evidence exists regarding the use of antipsychotics in patients with early-onset schizophrenia, which lags significantly behind studies on adult patients’ medication and reflects a large disparity from actual clinical needs. Thus, exploring the current status of drug treatment for early-onset schizophrenia may be helpful in choosing reasonable medications in clinical practice. Hence, this study aims to provide references on clinical medication for children and adolescents by conducting a retrospective analysis of the drug regimens of 746 inpatients with early-onset schizophrenia to analyse the status of drug use and its changes.

## Methods

### Study sample

Data were collected from hospitalization medical records in the Child Mental Health Research Center of the Affiliated Brain Hospital of Nanjing Medical University from March 2012 to July 2019. Inclusion criteria were as follows: 1) meeting the diagnostic criteria for schizophrenia per the International Classification of Diseases, 10th Edition (ICD-10), 2) age ≤ 18, and 3) hospital stay longer than 7 days. During hospitalization, three-level rounds were strictly performed to make a definitive diagnosis and establish treatment plans.

### Procedure

The patients’ medical history data were collected via the electronic medical record system, and the patients’ age, sex, diagnosis, days of hospitalization, family history of mental illness, drug prescription on the day of discharge, age of onset, duration of illness, PANSS total, positive and negative scores at admission, and other information were collected. All patients were clinically assessed within the first 24 h after their admission to the unit and on the day of discharge, by the staff, who had received training in the use of the PANSS. The efficacy outcome was assessed by the Clinical Global Impression-Global Improvement (CGI-I) scale [10] on the day of discharge. CGI-I scores range from 1 to 7 (with 1 being “very much improved”, 4 being “no change”, and 7 being “very much worse”). Much improvement was defined as clinical symptoms completely or basically having disappeared, and insight is partially restored. Improvement was defined as relief of clinical symptoms. No response was defined as no improvement or worsening of clinical symptoms. A score of one on the CGI-I was regarded as much improved, scores of two and three as improvement, and other scores as no response. To compare the treatment regimens of patients with schizophrenia at different ages, the patients were divided into an adolescent-onset schizophrenia (AOS) (i.e., psychotic symptoms appearing between the ages of 13 and 18) group and a child-onset schizophrenia (COS) (i.e., psychotic symptoms appearing before the age of 13) group. Approval for the study was granted by the Medical Ethics Committee of Nanjing Brain Hospital (2020-KY103–01), and the informed consent was waived by the Medical Ethics Committee.

### Statistical analysis

The statistical analyses were carried out using SPSS Statistics (v22.0 software). The measurement data were expressed by means ± standard deviations or medians, and the differences among groups were analysed by the *χ2* tests. *P* < 0.05 (two-tailed) was used to define differences that were statistically significant.

## Results

### Demographic and clinical characteristics of the study population

A total of 746 inpatients were included in the analysis. The basic demographic information and clinical characteristics of the patients are shown in Table 1. Among them, 40.1% of patients were male and 59.9% of patients were female. The mean age at admission was 13.98 ± 2.32 years. Six hundred and forty-one (85.6%) patients were experiencing first onset, with the mean age of onset of 12.94 ± 2.28 years, including 253 (33.9%) COS patients and 493 (66.1%) AOS patients. The mean duration of illness was 12.03 ± 13.37 months. The mean PANSS total, positive and negative scores at admission were 84.81 ± 19.67, 21.21 ± 5.56, and 19.57 ± 7.05, respectively. Of those patients who initiated drug treatment, 245 (32.8%) patients achieved considerable improvement and 495 (66.4%) patients achieved improvement. In only 6 (0.8%) patients did treatment prove to be no response.

**Table 1:** Demographic and clinical characteristics of the study population

	N (%)
**Demographic variables**	
Nanjing	132 (17.7%)
Male	299 (40.1%)
Age at admission, years	13.98 ± 2.32
**Clinical characteristics**	
Days of hospitalization	54.52 ± 32.28
First onset	641 (85.6%)
Refractory cases	224 (30.0%)
Age of onset, years	12.94 ± 2.28
Age < 13	253 (33.9%)
13 ≤ age ≤ 18	493 (66.1%)
Duration of illness, months	12.03 ± 13.37
IQ^a ^at admission	84.84 ± 0.85
PANSS total at admission	84.81 ± 19.67
PANSS positive at admission	21.21 ± 5.56
PANSS negative at admission	19.5 7 ± 7.05
Body weight change from admission to discharge, kg	1.00
**Efficacy**	
Much improvement	245 (32.8%)
Improvement	495 (66.4%)
No response	6 (0.8%)

Data were expressed by N (%), median or mean ± standard deviation

*Abbreviation: PANSS* Positive and Negative Symptoms Scale

^a^IQ was assessed by the Wechsler Child’s Intelligence Scale Revised (WISC-CR) for Chinese

### Distribution of types of medication on the day of discharge

#### Medication distribution on the day of discharge

On the day of discharge, 744 (99.7%) of the 746 patients were prescribed psychotropic drugs. Among all patients, 315 (42.2%) patients used anti-tremor paralysis drugs such as trihexyphenidyl, 66 (8.8%) used liver-protecting drugs such as polyene phosphatidylcholine capsules and silibinin, and 79 (10.6%) used heart-protecting drugs.

Seven hundred and forty-three (99.6%) patients were prescribed antipsychotics, involving 12 different types. Among them, 741 patients (99.3%) were prescribed atypical antipsychotics, and 41 patients (5.5%) were prescribed typical antipsychotics. The top five most frequently used antipsychotics were aripiprazole, olanzapine, risperidone, paliperidone and clozapine. An analysis of the frequency of use and the dosage of common antipsychotic drugs is shown in Table 2. In addition, 34 patients (4.6%) received mood stabilizers, 16 patients (2.1%) received antidepressants, and 66 patients (8.8%) received anti-anxiety drugs.

**Table 2:** Analysis of the use frequency and the dosage of commonly used antipsychotics

	N (%)	Dosage (mg/d)	Dosage range (mg/d)
Aripiprazole	247 (33.1%)	17.22 ± 7.14	2.50–30.00
Olanzapine	246 (33.0%)	17.29 ± 5.17	5.00–30.00
Risperidone	191 (25.6%)	3.57 ± 1.23	0.50–7.00
Paliperidone^a^	116 (15.5%)	8.33 ± 2.20	3.00–12.00
Clozapine	53 (7.1%)	140.64 ± 88.73	12.50–450.00

Data were expressed by N (%) or mean ± standard deviation

^a^Four cases using paliperidone injection (Invega Sustenna) were not included in the dosage analysis

#### The status of combination uses of antipsychotics

A total of 172 patients (23.1%) received a combination of antipsychotics, all of which were a combination of two types of antipsychotics. The common combinations of antipsychotics are shown in Table 3. The combination of aripiprazole and olanzapine was that most commonly seen in the AOS group, and the combination of risperidone and clozapine was that most commonly used in the COS group (Table 3).

**Table 3:** Analysis of the common combination of antipsychotic drugs

	Overall (172)	AOS (122)	COS (50)
Aripiprazole + Olanzapine	28 (16.3%)	25 (20.5%)	3 (6.0%)
Aripiprazole + Risperidone	20 (11.6%)	14 (11.5%)	6 (12.0%)
Risperidone + Clozapine	17 (9.9%)	10 (8.2%)	7 (14.0%)
Olanzapine + Risperidone	16 (9.3%)	10 (8.2%)	6 (12.0%)
Aripiprazole + Paliperidone	14 (8.1%)	13 (10.7%)	1 (2.0%)

### Related factors affecting the use of antipsychotics

#### Antipsychotics use in patients of different genders and types

Olanzapine and risperidone were used more frequently in men than in women, whereas aripiprazole was used less frequently in men. Olanzapine and paliperidone were used more frequently in AOS patients, and risperidone was used more frequently in COS patients (Table 4).

**Table 4:** Comparison of commonly used antipsychotics among different genders and types of patients

	Gender		*χ2*	*P*	Type		*χ2*	*P*
	Male (299)	Female (447)			AOS (493)	COS (253)		
Aripiprazole	52 (17.4%)	195 (43.6%)	55.669	0.001**	173 (35.1%)	74 (29.2%)	2.577	0.108
Olanzapine	118 (39.5%)	128 (28.6%)	9.507	0.002**	179 (36.3%)	67 (26.5%)	7.304	0.007**
Risperidone	96 (32.1%)	95 (21.3%)	11.081	0.001**	98 (19.9%)	93 (36.8%)	25.012	0.001**
Paliperidone ^a^	51 (17.1%)	65 (14.5%)	0.863	0.353	86 (17.4%)	30 (11.9%)	3.974	0.046*
Clozapine	26 (8.7%)	27 (6.0%)	1.914	0.167	32 (6.5%)	21 (8.3%)	0.830	0.362

Data were expressed by N (%) or mean ± standard deviation; statistic values were expressed by Pearson’s χ2 tests. *P < 0.05; **P < 0.01

^a^Four cases using paliperidone injection (Invega Sustenna) were not included in the dosage analysis

#### Trends in the use of antipsychotics in different years

**Fig. 1:**
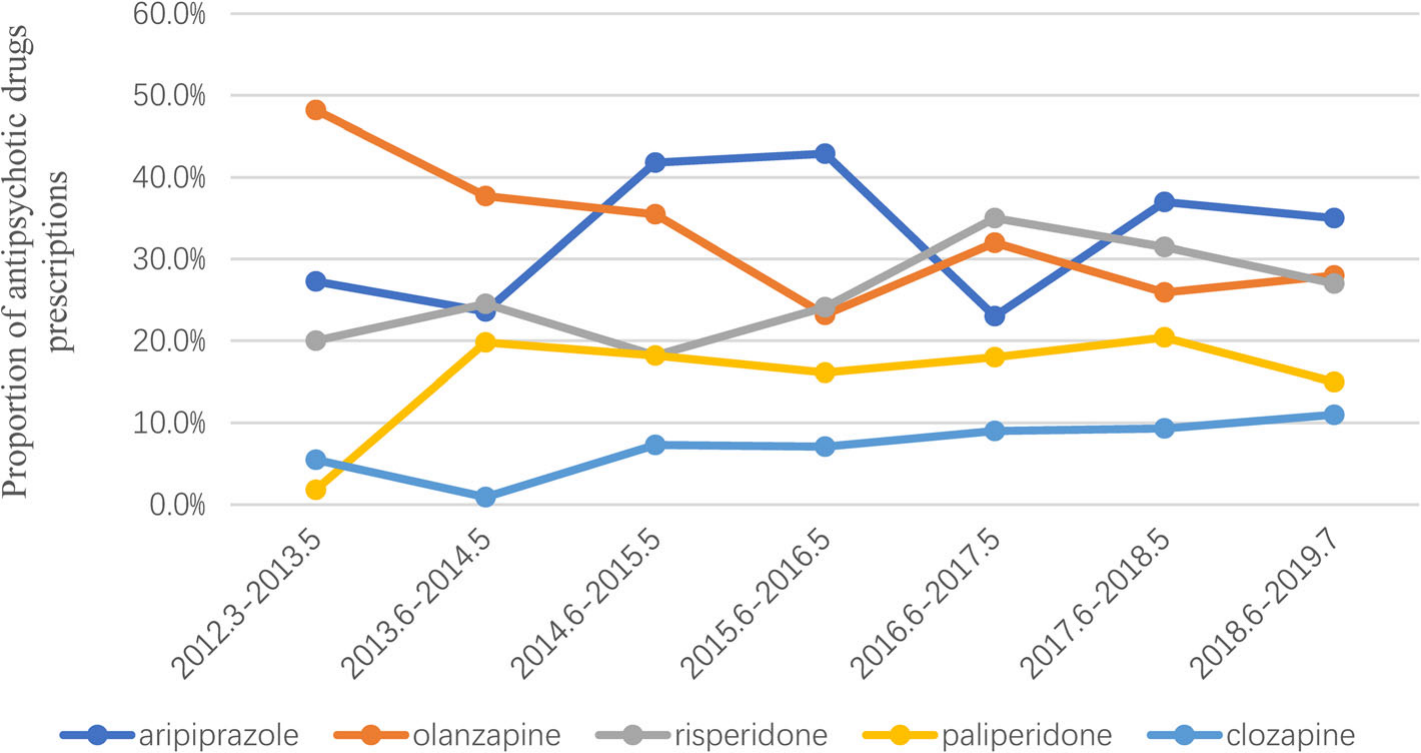
Trends in the proportion of prescriptions of commonly used antipsychotics in the past 7 years

In the past 7 years, the frequency of use of clozapine has been on the rise. The frequency of use of olanzapine has been less than in previous years. The use of aripiprazole, risperidone, and paliperidone has fluctuated (Fig. 1.). Paliperidone and aripiprazole were approved by the China Food and Drug Administration (CFDA) for the treatment of psychosis in children and adolescents in September 2017 and January 2018, respectively. There was no significant difference in the frequency of use of these two drugs before and after CFDA approval (Table 5).

**Table 5:** Comparison of the use of paliperidone and aripiprazole in children and adolescents before and after CFDA’s approval

	Before CFDA approval	After CFDA approval	*χ2*	*P*
Aripiprazole prescription, %	30%	34.1%	0.543	0.461
paliperidone prescription, %	18%	15.6%	0.303	0.582

## Discussion

In this study, almost all inpatients were prescribed atypical antipsychotics, and the top five most commonly used antipsychotics were aripiprazole, olanzapine, risperidone, paliperidone and clozapine. A combination of antipsychotics was prescribed in 23.1% of patients. The combination of aripiprazole and olanzapine was that most commonly seen in the AOS group, and the combination of risperidone and clozapine was that most commonly used in the COS group. Olanzapine and risperidone were used more frequently in men, whereas aripiprazole was used less frequently. Olanzapine and paliperidone were used more frequently in AOS patients, and risperidone was used more frequently in COS patients.

In this study, female patients accounted for 59.9% of patients, which seems to be unusual. The reason may be that the older male patients with obvious impulsive and aggressive behaviours may have been admitted to the adult ward in our hospital. The mean age of onset was 12.94 ± 2.28 years, and patients with COS accounted for 33.9% of the sample. The younger the age of onset is, the more difficult the treatment is [11]. Children and adolescents are different from adults in terms of physiology, anatomy, developmental maturity, and response and tolerance to drugs. The same patient has non-linear differences in drug clearance and metabolic capacity at different growth stages. Dosage, frequency, etc. will have be significantly impacted. Children have strong metabolisms and are more sensitive to drugs than adults, and they may be more susceptible to the side effects of drugs than adults [12]. Therefore, the choice of drugs is critical, and the adjustment of medication needs to be timely and accurate [13].

This study found that almost all patients were prescribed second-generation antipsychotics (SGAs), with only 5.5% of patients prescribed with first-generation antipsychotics (FGAs). The use of antipsychotics (APs), especially SGAs, is on the rise in the paediatric population worldwide [14, 15]. This may relate to the fact that most SGAs have slightly adverse effects on children and adolescents in terms of safety and pharmacokinetics [13, 16]. For instance, a meta-analysis [6] showed that aripiprazole, olanzapine, risperidone, and paliperidone and quetiapine had significantly better efficacy than the placebo for EOS patients. Clozapine (only for refractory patients) was the most effective. In addition, the acceptability of paliperidone, risperidone, olanzapine and quetiapine was higher than that of the placebo. Considering the efficacy and safety of aripiprazole, olanzapine, risperidone and paliperidone, the Food and Drug Administration (FDA) has approved these four drugs in the treatment of mental illness in children and adolescents, among which paliperidone and aripiprazole were also approved by the CFDA for the treatment for mental disorders in children and adolescents in September 2017 and January 2018, respectively. Despite the efficacy of APs having been proven, its use in children and adolescents still faces compounding challenges. An unmet challenge is the side effects caused by atypical antipsychotics, especially metabolic syndrome [17, 18]. Rates of metabolic problems are higher in young first-episode patients [19], and children and adolescents appear to be at greater risk of weight gain from antipsychotics than adults [20]. The effects of induced weight gain for atypical antipsychotic drugs vary, with olanzapine having the strongest effect; moreover, typical antipsychotic drugs may also cause significant weight gain, among which chlorpromazine has the strongest effect [21]. In conclusion, children and adolescents are at a developmentally sensitive stage of life; thus, the balance between the need for symptom improvement and drug-induced adverse reactions should be considered when prescribing antipsychotics.

The second challenge is a lack of evidence that antipsychotics improve cognitive function. Cognitive dysfunction is the core symptom of schizophrenia [22]. Studies have shown that schizophrenia patients suffer from impairments in processing speed, working memory, social cognition and other areas [23]. Recent longitudinal studies have also shown that poor cognitive ability seems to be associated with consistently poor functional outcomes [24]. Cognitive function is of great concern to children and adolescents. However, the effects of antipsychotics on cognitive function are not yet fully understood. This lack of clarity exists despite research showing that SGAs can improve cognitive performance [25, 26]. However, the effect size is small [27]. Some studies have found that the cognitive improvement observed in first-episode schizophrenia may be due to exercise effects (e.g., exposure, familiarity, and/or programmed learning) [28]. Furthermore, metabolic problems secondary to atypical antipsychotics are common, which are associated with cognitive impairment [29]. That is, the metabolic side effects of antipsychotics may exacerbate cognitive impairment [30]. Excessive dopamine blockade has been linked to cognitive impairment [31]. Recent studies have shown that cognitive function can be improved by reducing the dose of risperidone and olanzapine, and mechanistically, the cognitive improvement that occurs after dose reduction of an antipsychotic may be the result of decreased dopamine D2 receptor blockade [32].

The third challenge is that most available antipsychotics have limited effects on negative symptoms and are studied almost exclusively in adults. Negative symptoms are associated with long-term poor prognosis [33]. Some studies have shown that certain antipsychotics can relieve negative symptoms. For example, a meta-analysis [34] showed that 18 antipsychotics significantly reduced negative symptoms compared to placebo, with clozapine, amisubril, olanzapine, levotipine and risperidone (to a lesser extent) significantly reducing negative symptoms more than others. A prospective, large-scale, randomized trial [35] compared the efficacy of fixed doses of cariprazine and risperidone in the treatment of negative symptoms. In this 26-week study, cariprazine was superior to risperidone in terms of negative symptoms and function improvement. However, the effect of cariprazine on negative symptoms in children and adolescents has not been studied. A combination of antipsychotics and antidepressants may also improve negative symptoms while addressing some mood disorders associated with schizophrenia [36]. However, there is no clear information on the effect of this combination on the negative symptoms or on the mechanism of action. Patients, especially children and adolescents, rarely complain of negative symptoms, and more pressing positive symptoms may distract the clinician [37]. Clinicians should carefully monitor and actively manage all clinically relevant negative symptoms. Drug development targeting negative symptoms has yielded positive results for selected monotherapies in a handful of recent well-designed clinical trials, but effective treatment of negative symptoms remains an unmet medical need for schizophrenia to date.

In this study, the antipsychotics selected for AOS and COS patients were different. For example, olanzapine and paliperidone were used more frequently in AOS patients than COS patients. The possible reasons could be the short onset time and stronger sedative effect of olanzapine, which makes it more conducive to controlling impulsive behaviours and positive symptoms of schizophrenia. In addition, olanzapine can relieve symptoms quickly in the acute phase of psychosis [38]. Paliperidone is a new type of monoamine antagonist, whose major ingredient is 9-hydroxyrisperidone—the active metabolite of risperidone. It is made into to a sustained-release dosage form, which delays the drug-release process and ensures a stable blood-drug concentration; it also lowers the risk of liver damage mainly due to renal excretion and reduces drug interaction, which may offer important clinical advantages. For patients with early-onset schizophrenia, taking medication once a day helps maintain the patients’ compliance. Moreover, paliperidone has less of an effect on liver function, which is much more easily accepted by children and adolescents, who are more sensitive to drug side effects [39, 40]. Risperidone was more commonly used in patients with COS. An open controlled study abroad showed that risperidone can significantly improve the positive and negative symptoms of adolescent schizophrenia patients and cause less extrapyramidal reactions than traditional antipsychotics [41, 42]. The frequencies of use of aripiprazole and clozapine were not significantly different in the AOS and COS groups. When taking aripiprazole, extrapyramidal syndrome, hyperprolactinemia, weight gain, metabolic disorders, sedation and other side effects are rarely seen. A randomized controlled study showed that adolescents treated with aripiprazole had a significantly longer time to exacerbation or relapse than the placebo group [43]. It can be seen that aripiprazole is safe and effective for the treatment of early-onset schizophrenia. Meanwhile, aripiprazole can also safely treat hyperprolactinemia caused by other antipsychotics [44]. Thus, aripiprazole is more suitable for female patients, which is consistent with the results of this study. Olanzapine was used more frequently in men in this study, which may be related to the fact that men are less troubled by endocrinology.

In this study, 23.1% of patients were prescribed multiple antipsychotics during hospitalization, of which aripiprazole and olanzapine were the most commonly used combination in the AOS group and risperidone and clozapine were the most commonly used in the COS group. In clinical practice, it is common to use multiple antipsychotic drugs during the treatment of EOS, although this is not recommended by guidelines. In this study, refractory cases accounted for approximately 30%. It has been found in clinical practice that treatment for refractory schizophrenia often requires combination treatment. Clozapine is often used jointly with other drugs, in which the dose of clozapine could be appropriately lowered to reduce the rate of adverse clozapine reactions and increase the patient’s compliance with treatment [45]. Tiihonen, et al. [46] have studied the relationship between the drug combination therapy for psychosis and psychosis relapse; they found that, in general, any antipsychotic combined with others can reduce the risk of psychiatric readmissions by 7–13% compared with single-drug treatment, among which the combination of aripiprazole and clozapine is associated with the lowest risk of readmission. This seems to be contrary to the current treatment guidelines advocating the principle of single-drug treatment. However, the truth is that the treatment of patients with EOS is difficult, and there are many refractory cases; thus, multiple drug treatments are often required [45, 47]. Apart from the combined use of antipsychotics, the treatment of schizophrenia often also includes the combination of antipsychotic drugs and other psychotropic drugs. In this study, 8.8, 4.6, and 2.1% of patients used antipsychotic drugs with anti-anxiety drugs, mood stabilizers, and anti-depressant drugs, respectively. Stroup, et al. [48] found that the addition of antidepressant drugs was associated with a significant reduction in the hospitalization rate of adult schizophrenic patients compared with the addition of another antipsychotic drug. Although the addition of mood stabilizers was not significantly related to the risk of hospitalization, it did increase the risk of death. Thus, the combination of psychotropic drugs still needs to be applied cautiously.

In psychiatric prescriptions for children and adolescents, off-label use is a common and important problem due to the small variety of drugs available. Most antipsychotic drugs are developed by foreign manufacturers and have been approved by the FDA before they are marketed in China. Therefore, clinicians in China are likely to refer to foreign guidelines and medication instructions when using these drugs in clinical diagnosis and treatment before they are approved by CFDA [49]. In this study, we can see that paliperidone and aripiprazole were stably used before and after being approved by the CFDA for clinical use, which also confirms that clinical practice often precedes the development of guidelines, and practical experience can contribute to the development of guidelines as well. The study showed that the use of olanzapine has been less than that seen in previous years, which may result from the approval of paliperidone and aripiprazole for clinical use in China, and clinicians are also increasingly considering adolescents to be at high risk for developing metabolic syndrome. Of course, it is necessary to pay attention to the right of informed consent of patients and their families in clinical work; particularly in the case of off-label use, patients and their families should be fully informed and consenting, and they should sign the informed consents for the drug’s use.

There were several limitations to this study. First, the results of the present study are limited by its retrospective design rather than longitudinal study and this may be the case in most of previous studies concerned with retrospective data. Furthermore, the results of this study may be limited in Chinese population.

## Conclusions

In summary, the present study retrospectively analysed the clinical drug use of patients with early-onset schizophrenia in our ward over the past 7 years, indicating that atypical antipsychotics have been increasingly valued and used clinically. Clinicians should select the appropriate medication regimens based on the age, sex, metabolism and cognitive function at baseline of patients with early-onset schizophrenia. Although the guidelines emphasize a single medication, the combination of several antipsychotics is clinically meaningful in the treatment of refractory patients. Only with reasonable and scientific consideration of the medication regimens can children and adolescents with schizophrenia recover fully and quickly.

## Data Availability

The datasets generated and/or analyzed during the current study are not publicly available due reason why data are not public but are available from the corresponding author on reasonable request. Additionally, any further permission from the hospital is required to access the medical records of patients.

## Abbreviations

EOS: Early-onset schizophrenia
COS: Child-onset schizophrenia
AOS: Adolescence-onset schizophrenia
PANSS: Positive And Negative Syndrome Scale
CGI-I: Clinical Global Impression-global improvement (CGI-I)
SGAs: The second-generation antipsychotics
FGAs: The first-generation antipsychotics
Aps: Antipsychotics
FDA: Food and Drug Administration
CFDA: China Food and Drug Administration
APZ: Aripiprazole
TDM: Therapeutic drug monitoring
